# The influence of xeno-free culture conditions on the angiogenic and adipogenic differentiation properties of adipose tissue-derived stem cells

**DOI:** 10.1016/j.reth.2024.09.013

**Published:** 2024-10-10

**Authors:** Anne Therese Lauvrud, Maria Vittoria Giraudo, Rebecca Wiberg, Mikael Wiberg, Paul J. Kingham, Maria Brohlin

**Affiliations:** a Department of Medical and Translational Biology, Umeå University, SE-901 87 Umeå, Sweden; b Department of Diagnostics and Intervention, Umeå University, SE-901 87 Umeå, Sweden; c Department of Clinical Microbiology, Umeå University, SE-901 87 Umeå, Sweden

**Keywords:** Cell-assisted lipotransfer, Mesenchymal stem cells, Regenerative medicine, Stem cell therapy, Xeno-free

## Abstract

**Introduction:**

Before performing cell therapy clinical trials, it is important to understand how cells are influenced by different growth conditions and to find optimal xeno-free medium formulations. In this study we have investigated the properties of adipose tissue-derived stem cells (ASCs) cultured under xeno-free conditions.

**Methods:**

Human lipoaspirate samples were digested to yield the stromal vascular fraction cells which were then seeded in i) Minimum Essential Medium-α (MEM-α) supplemented with 10 % (v/v) fetal bovine serum (FBS), ii) MEM-α supplemented with 2 % (v/v) human platelet lysate (PLT) or iii) PRIME-XV MSC expansion XSFM xeno-free, serum free medium (XV). Flow cytometry for ASCs markers CD73, CD90 and CD105 together with the putative pericyte marker CD146 was performed. Growth rates were monitored over multiple passages and adipogenic differentiation performed at early and expanded passage culture. Growth factor gene expression was analyzed and an *in vitro* angiogenesis assay performed.

**Results:**

Cells in FBS and PLT grew at similar rates whereas the cells cultured in XV medium proliferated significantly faster up to 60 days in culture. All cultures were >98 % positive for CD73, CD90 and CD105, whereas CD146 expression was significantly higher in XV cells. Adipogenic differentiation was most pronounced in cells which had been cultured in XV medium whilst cells grown in PLT were inferior compared with cells from the FBS cultures. *IGF1* gene expression was highest in cells cultured in PLT whilst cells grown in XV medium showed 10-fold lower expression compared with FBS cells. In contrast, *HGF* gene expression was 90-fold greater in cells cultured in XV medium compared with those cultured in FBS. Conditioned medium from XV cultured cells showed the most angiogenic activity, inducing the greatest endothelial cell network formation and maturation.

**Conclusion:**

Culture under different conditions alters the ASCs characteristics. Since cells cultured in XV medium showed the best adipogenic and angiogenic profile this might be a preferred medium formulation for preparing cells required for reconstructive surgical applications such as cell-assisted fat grafting.

## Highlights

• Enhanced proliferation of adipose tissue-derived stem cell in xeno-free medium.
• Optimal adipogenic differentiation of xeno-free medium expanded stem cells.
• High angiogenic activity associated with xeno-free cultured stem cells.

## 1 Introduction

Human adult mesenchymal stem cells are multipotent stem cells that can be isolated from various tissues including fat [1]. Adipose tissue-derived stem cells (ASCs) are defined by various properties such as adherence to tissue culture plastic, expression of surface markers (CD73, CD90 and CD105) and differentiation towards adipo-, osteo- and chondrogenic lineages [2,3]. Many studies have shown the regenerative properties and anti-inflammatory effects of adult stem cells, with numerous ongoing clinical trials regarding stem cell therapy throughout the world [4,5].

Fat grafting is a popular method in reconstructive surgery [6]. ASCs found within the grafts have not only the capacity to differentiate into new fat cells, but also aid the vascularization of the graft and reduce tissue necrosis whilst also recruiting distant stem cells to the repair site [[6], [7], [8]]. Nevertheless, fat grafting is an unpredictable technique with highly variable graft survival outcomes [9]. One way to overcome this problem is to enrich the fat grafts with stromal vascular fraction (SVF), in a process known as cell-assisted lipotransfer [10]. SVF enrichment can simply be done inside the operation theatre and is not classified as a regulated form of cell therapy. The SVF is a heterogenous mixture of cells that includes the ASCs and other regenerative cell types [11]. Studies have shown that this procedure may improve the survival of the fat due to better angiogenic and adipogenic conditions in the graft site but, as is the case for fat grafting alone, results are highly variable [12]. In some cases, for example in radiated tissues or large volume fat grafting there is a need for a higher number of cells and ideally use the most potent regenerative cells to enhance the survival of the graft. We have previously identified that a CD146 selected population of ASCs (putative pericytes) show enhanced adipogenic and angiogenic properties which might thus be of benefit for such applications [13].

To obtain a sufficient number of cells requires culture and expansion of the cells *in vitro* prior to injection into patients. When there is a need for culture expansion, the procedure must be safe and devoid of any animal products (namely xeno-free) [14] and requires that the cells should be produced in accordance with Good Manufacturing Practice (GMP) [15]. Traditionally, fetal bovine serum (FBS) has been used as a supplement for the expansion of ASCs *in vitro* [16]. However, FBS contains animal-derived components that can potentially cause adverse reactions in humans, making it unsuitable for use in clinical treatments [4]. Previous studies have shown that the safety and function of the ASCs can be strongly influenced by the composition of the culture media [17,18]. Thus, today various xeno-free defined media formulations have been developed and are undergoing investigation [17]. Another way to grow and culture cells is in human platelet lysate (PLT) which is added as a growth supplement to basal media formulations and has been shown to have positive effects on different types of cells and indications. Pooling of mixed human platelet lysate units into one PLT batch can balance donor variation with regard to essential platelet-derived growth factors and cytokines [19,20]. Nevertheless, using a defined xeno-free medium is likely to be more favorable for large scale cell culture expansion due to its lot-to-lot consistency.

In summary, when developing culture conditions with view to clinical applications there is a need to find the optimal balance between the rate of cell growth and differentiation potential with reproducibility and consistency whilst also addressing regulatory concerns and final costs of production. Therefore in this study our aim was to evaluate and characterize ASCs cultured in PLT and a commercially available defined xeno- and serum-free medium (PRIME-XV MSC expansion XSFM (FujiFilm, Irvine Scientific)) and compare them with culture in the presence of the traditional supplement of FBS. The chosen defined medium was selected on the basis of its reported ability to support ASCs expansion, manufacture under cGMP and the availability of drug master file registration. We focused on investigating the effects on the angiogenic and adipogenic properties of the ASCs with a view to their potential applications in reconstructive surgery such as cell-assisted fat grafting.

## 2 Materials and methods

### 2.1 Isolation of adipose tissue-derived stem cells

Human adipose tissue samples from 7 female donors (mean age 49.5 years, mean BMI 27.5) obtained as waste material after liposuction procedures performed at Umeå University Hospital were used to isolate the SVF containing the ASCs. Written informed consent was received and procedures were approved by the Local Ethical Committee for Clinical Research in Umeå University (2013-276-31 M). The samples were digested with collagenase NB4 (Serva/Nordmark) dissolved in saline solution at a concentration of 0.3U per ml of aspirate. Samples were incubated for 1 h at 37 °C and were then centrifuged at 300 g for 5 min. The undigested fat tissue and collagenase solution was aspirated, and the pellet was resuspended in saline. Red blood cells were lysed with ACK (Ammonium-Chloride-Potassium) Lysing Buffer (ThermoFisher Scientific), the samples re-centrifuged and the resulting pellet resuspended in either Minimal Essential Medium-α (MEM-α) containing 10 % (v/v) fetal bovine serum, MEM-α containing 2 % (v/v) human platelet lysate (PLTGold® Human Platelet Lysate; Millipore) or PRIME-XV MSC Expansion XSFM medium (FujiFilm Irvine Scientific; hereafter referred to as the XV group) each together with 1 % (v/v) penicillin/streptomycin (Invitrogen Life Technologies). The first two groups were cultured on polystyrene flasks (Nunc® EasYFlask™) and the XV group on Corning® CellBIND® flasks. Cells were seeded at a minimum starting density of 10,000 cells/cm^2^ and subsequently incubated at 37 °C with 5 % CO_2_. The non-adherent cells were removed after 24 h by washing with saline solution, followed by replenishment with fresh growth medium in the three respective groups. On reaching approximately 90 % confluence the adherent cells were passaged by using a solution of TrypLE Express (Invitrogen). After trypsinization, cells were seeded in culture flasks at a density of 2000 cells/cm^2^ and allowed to expand up to 90 % confluency (6–7 days for the FBS and PLT groups and 4 days for the XV group) before re-trypsinization, counting and replating. Some samples were frozen in Synth-a-Freeze® Cryopreservation Medium (ThermoFisher) and stored at −80 °C for later use.

### 2.2 Populating doubling (PD)

Cells at each passage were counted under a microscope using a Neubauer 0,100 mm hemocytometer and the number of population doublings calculated according to the following equation:

n=3.32(logUCY−logI)+X

where n = the cumulative PD number at end of a given subculture, UCY = the cell yield at that point, I = the cell number used to begin the subculture, and X = the number of PD undergone by the cells used in the seeding. Data were plotted as cumulative population doublings.

### 2.3 Phenotypic characterization

To confirm the presence of ASCs derived from the SVF, ASCs at early passage (P1-2) and expanded passage (P7-8) were characterized by flow cytometry for positive mesenchymal stem cell-associated surface markers (CD73, CD90, CD105) and the putative pericyte marker CD146 using PE-conjugated antibodies (BD Biosciences). As a negative control, a corresponding isotype control was used for each sample (mouse IgG1, κ). The analysis was performed on cells from each donor, with a minimum of 10,000 cells chosen for each analysis. Data were acquired using a BD Accuri™ C6 flow cytometer (BD Bioscience).

### 2.4 Adipocyte differentiation

Early passage and expanded passage cells were treated with TryPLE, counted, and plated into 24-well CellBIND™ plates at a density of 55,000 cells/well. Before beginning the adipogenic differentiation the wells were checked for confluency. Medium was switched to adipogenic differentiation medium consisting of low glucose Dulbecco's Modified Eagle's Medium (DMEM) with 10 % (v/v) FBS, 1 % (v/v) penicillin/streptomycin solution plus 1 μM dexamethasone, 0.5 mM 3-isobutyl-1-methylxanthine (IBMX), 100 μM indomethacin and 10 μg/ml insulin (all from Merck/Sigma). The medium was changed every 2 days. On days 7 and 15 after beginning the induction, dexamethasone, IBMX and indomethacin were omitted from the medium. After 21 days the wells were stained with Oil Red O solution to confirm the presence of lipid droplets and photographs taken using an Olympus microscope and camera system. To quantify the Oil Red O, the samples were destained using 60 % isopropanol and absorbance measured at 492 nm using a Synergy Microplate reader. Samples for qRT-PCR analyses were also taken and cell-conditioned medium frozen for ELISA analyses. In some experiments the adipogenic differentiation was performed in medium containing 2 % (v/v) PLT.

### 2.5 Osteogenic and chondrogenic differentiation

To confirm multi-lineage differentiation, the standard culture FBS cells at passage 1 were plated into 24 well plates at a density of 55,000 cells/well after coating the wells with collagen and vitronectin overnight. Once the cultures were confluent, an osteogenic differentiation protocol was initiated and continued for a period of 21 days, before staining with Alizarin Red as previously described [13]. For chondrogenesis, 3D aggregate cultures were used. ASCs (250,000 cells) were resuspended in ChondroMAX Differentiation Media (Merck Millipore), centrifuged at 200 g for 5 min and the pellet resuspended with 0.5 ml ChondroMAX Differentiation Media. The cells were then re-centrifuged and thereafter with the medium remaining, the cap of the tube was loosened to allow gas exchange and the sample was incubated upright in an incubator at 37 °C with 5 % CO_2_. Once the cell pellet had formed a round ball, the medium was changed every other day. After 21 days the cells were fixed, sectioned (12 μm) and stained with 1 % (w/v) Toludine Blue.

### 2.6 Gene expression

Using an RNAeasy kit (Qiagen) RNA was isolated from the early and expanded passage undifferentiated cells and also the differentiated adipocytes. The RNA was converted into cDNA using an iScript™ cDNA synthesis kit (Bio-Rad). qRT-PCR was performed using SsoFast™ EvaGreen supermix (Bio-Rad) in a CFX96 Optical Cycler and analyzed using the CFX96 manager software (Bio-Rad) as described previously [13]. Alternatively, qRT-PCR was performed using Myra Liquid Handling System (Bio Molecular Systems) and Mic qPCR cycler (Bio Molecular Systems) with the SsoFast™EvaGreen® supermix (Bio-Rad). The primer sets used are shown in Table 1.

**Table 1:** qRT-PCR primer sequences and annealing temperatures (ºC).

Gene	Forward (5′-3′)	Reverse (5′-3′)	(ºC)
*AP2*	GGTGGTGGAATGCGTCATG	CAACGTCCCTTGGCTTATGC	65.0
*PPARG*	TACTGTCGGTTTCAGAAATGCC	GTCAGCGGACTCTGGATTCAG	62.6
*ANG*	TGGCAACAAGCGCAGCATCAAG	GCAAGTGGTGACCTGGAAAGAAG	68.2
*ANGPT1*	CTTGACCGTGAATCTGGAGC	AGCAAGACATAACAGGGTGAG	59.7
*HGF*	CAATAGTCAATTTAGACCATCCC	CGTGTTGGAATCCCATTTACAA	62.4
*IGF1*	TGTGGAGACAGGGGCTTTTA	ATCCACGATGCCTGTCTGA	61.5
*VEGFA*	ATCTGCATGGTGATGTTGGA	GGGCAGAATCATCACGAAG	61.4
*IL1B*	TTCGACACATGGGATAACGAGG	TTTTTGCTGTGAGTCCCGGAG	65.2
*TNF*	GTGACAAGCCTGTAGCCCAT	TATCTCTCAGCTCCACGCCA	62.1
*IL10*	GGAGAACCTGAAGACCCTC	ACTCACTCATGGCTTTGTAGAT	56.8
*MCP1*	AATAGGAAGATCTCAGTGCA	TCAAGTCTTCGGAGTTTGGG	57.3
*TGFB1*	GGCCAGATCCTGTCCAAGC	GTGGGTTTCCACCATTAGCAC	63.3
*RPL13A*	AAGTACCAGGCAGTGACAG	CCTGTTTCCGTAGCCTCATG	58.0

### 2.7 Enzyme-linked immunosorbant assay (ELISA)

Cell-conditioned medium was collected from adipogenic-differentiated cells at day 21 and analyzed for adiponectin using a sandwich ELISA kit (RayBiotech Inc). Absorbance measurements at 450 nm were made using a BioTek Synergy Microplate reader (Agilent Technologies) and concentrations of proteins calculated from standard curves.

### 2.8 Angiogenic assay

Cells were plated in their respective media at 100,000 cells per well in 24-well CellBIND™ plates and allowed to settle for 24 h. Thereafter, to eliminate possible high background contamination of growth factors in the respective culture media (especially the PLT supplemented samples), cells were washed twice in saline and then MEM-α medium alone was added to the wells. Cell-conditioned medium was collected after 48 h and applied to the angiogenesis assay. In brief, stock ECM Gel from Engelbreth-Holm-Swarm murine sarcoma was thawed overnight at 4 °C. Gel (175 μl) was transferred to individual wells in a 48-well plate and then the plates were transferred to the cell culture incubator to allow the gel to polymerize for 30 min at 37 °C. Thereafter 25,000 human umbilical vein endothelial cells (HUVECs; Invitrogen) were resuspended in the respective cell-conditioned supernatants and added on top of the gels. The HUVECs were allowed to attach for 2 h before tube formation was evaluated by light microscopy (Olympus IX71 microscope). A picture was taken in the middle of each well using a ColorViewII digital camera (Soft Imaging System) and analyzed using Image-Pro® Plus software (Media Cybernetics) for either total network length (continuously joined end–end cells) or the number of closed polygon-shaped structures which are representative of mature capillary networks. Triplicate wells were used for each condition and the experiment was repeated twice.

### 2.9 Statistical analysis

GraphPad Prism versions 8 and 10 (GraphPad Software Inc. Boston, USA) were used to perform statistical analyses on samples from 4 to 7 donors for the different types of experiments. Where appropriate, paired t-tests were performed or for multiple comparisons one-way ANOVA with Bonferonni post-hoc test was used. Statistical significance was determined as ∗P < 0.05, ∗∗P < 0.01 and ∗∗∗P < 0.001.

## 3 Results

### 3.1 Stem cell properties and proliferation

**Fig. 1:**
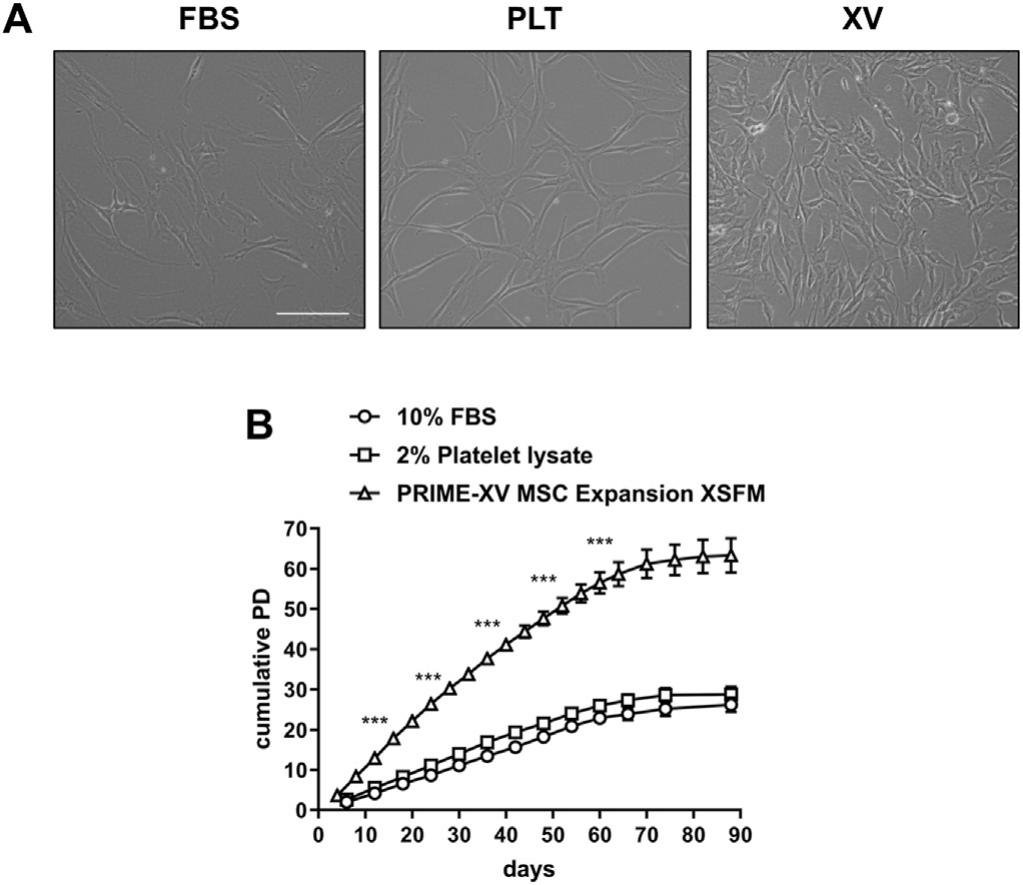
**Morphology and proliferation of ASCs**. **(A)** Representative phase-contrast images of cells cultured in FBS, PLT or Prime-XV MSC Expansion XSFM medium (XV). **(B)** Cumulative population doublings (PD) of ASCs-cultured in FBS, PLT or XV medium during 90 days. Results are shown as mean ± s.e.m from 7 donors. ∗∗∗ represents p < 0.001 significantly different XV versus FBS/PLT at the selected correspond time points indicated. Scale bar = 50 μm.

**Fig. S1:**
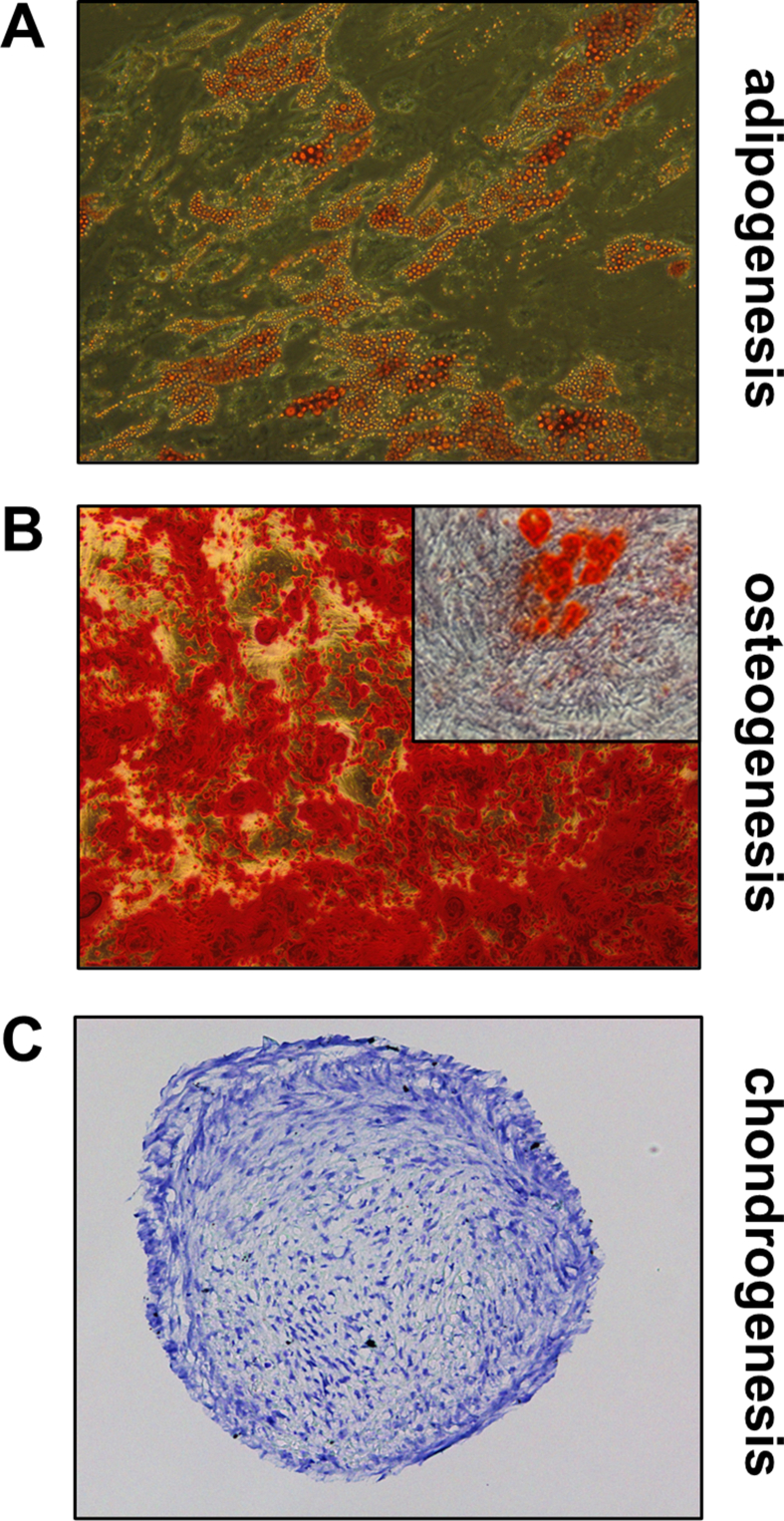
Tri-lineage differentiation of ASCs. Cells were treated with (A) adipogenic medium (B) osteogenic medium or (C) chondrogenic medium. Cells were respectively stained with Oil Red O, Alazarin Red and Toluidine Blue. Inset in B shows a donor with minimal osteogenic differentiation. Main images shown from one corresponding donor.

After isolation, SVF cells were immediately plated in MEM-α medium containing 10 % (v/v) FBS, MEM-α medium containing 2 % (v/v) PLT or in XV medium and allowed to reach approximately 90 % confluence before replating for population doubling counts with multiple passaging and replating of the ASCs. The morphology of cells in the presence of FBS or PLT were similar although the latter group tended to be more elongated/bipolar shaped (Fig. 1A). The cells cultured in XV medium were much smaller with finer filopodia-like processes (Fig. 1A). Cells under all culture conditions were expanded through 90 days *in vitro* at which point growth plateaued. Cells in FBS and PLT grew at similar rates whereas the cells cultured in XV medium proliferated significantly faster with 4–5 population doublings every 4 days up to approximately 60 days whereafter growth rates began to slow and finally plateau (Fig. 1B). Standard condition cells (FBS expanded) were tested for tri-lineage differentiation to confirm stemness. Under differentiating conditions all donors efficiently differentiated into adipocytes (Oil Red O positive lipid staining) whereas there were variable levels of osteogenic (Alazarin Red positive mineral staining) and chondrogenic (Toluidine Blue positive proteoglycan staining) differentiation (Supplemental Fig. 1).

**Fig. 2:**
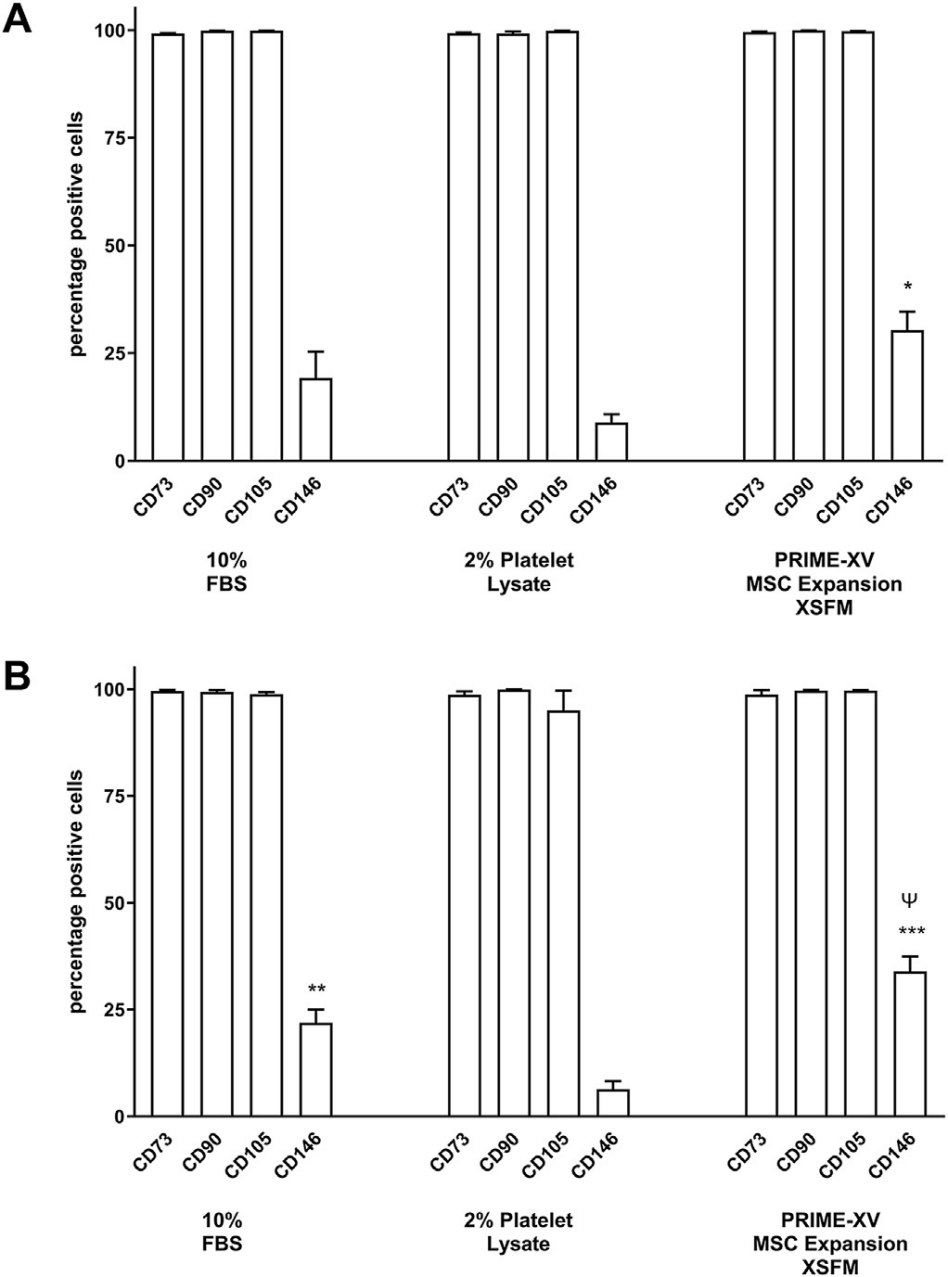
**Flow cytometry analyses of ASCs-associated surface marker expression.** The expression levels of CD73, CD90, CD105 and CD146 were analyzed at **(A)** early (P1-2) and **(B)** expanded passage (P7-8) in the ASCs cultured in medium supplemented with FBS or PLT or in Prime-XV MSC Expansion XSFM medium (XV). The CD marker expression levels are illustrated in histograms as percentage of positive cells from 7 donors. The histograms show mean ± s.e.m. ∗ Represents p < 0.05, ∗∗p < 0.01 and ∗∗∗p < 0.001 significantly different compared with expression levels in PLT cultured cells. Ψ represents ∗ p < 0.05 significantly different compared with expression levels in cells grown in medium containing FBS.

All cultures were >98 % positive for the mesenchymal stem cell markers CD73, CD90 and CD105. In contrast a smaller fraction of the cells were positive for the pericyte marker CD146. Early passage cultures in XV medium showed 30 ± 4 % positive CD146 expression which was significantly (P < 0.05) greater than cultures in PLT (9 ± 2 %). Early passage cultures in FBS contained 19 ± 6 % CD146 positive cells (Fig. 2). Similar expression levels were observed in cultures expanded for multiple passages with significantly higher CD146 expression in XV medium versus PLT (P < 0.001) and versus FBS (P < 0.05) (Fig. 2).

### 3.2 Adipogenic differentiation

**Fig. 3:**
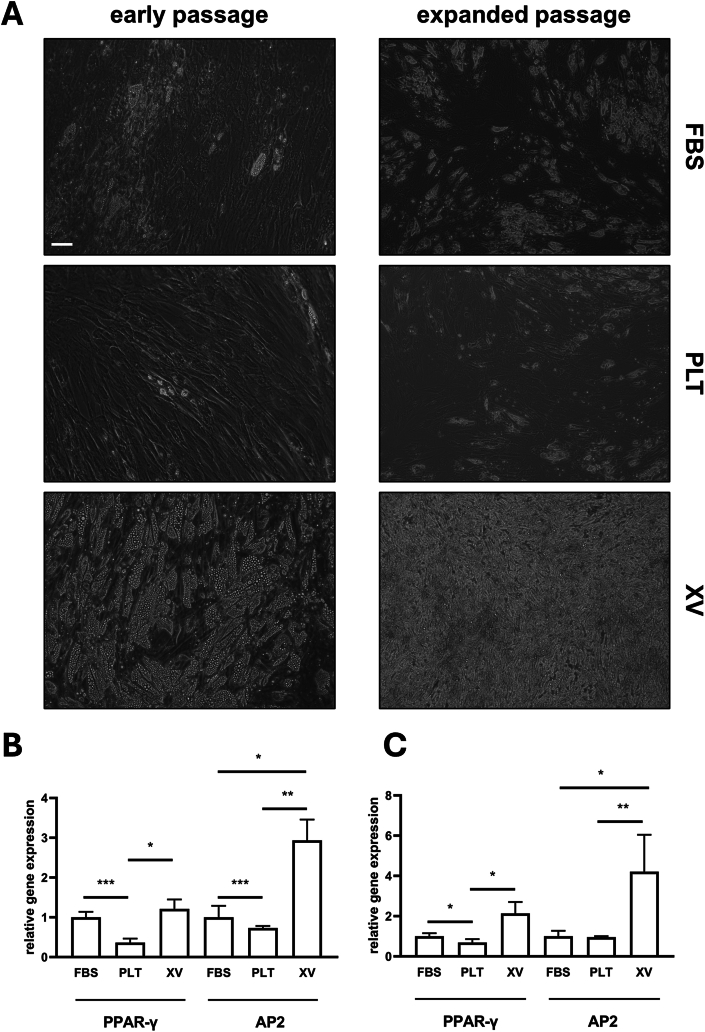
Adipogenic differentiation of ASCs and analysis of adipogenic gene expression. (A) Phase contrast images of cells which had been cultured in medium containing FBS or PLT or in Prime-XV MSC Expansion XSFM medium (XV) after exposure to adipogenic differentiation medium at day 11. Representative images from one donor are shown. Scale bar = 30 μm. The gene levels for *PPAR-gamma* and *AP2* in the adipogenically differentiated cells from early passage (B) and expanded passage (C). qRT-PCR was performed on samples from 5 to 7 donors. The histograms show mean ± s.e.m gene expression levels relative to cells which had been cultured in medium containing FBS. ∗ Represents p < 0.05, ∗∗p < 0.01 and ∗∗∗p < 0.001 significant differences between the groups shown by connecting lines.

ASCs were differentiated towards adipocytes. At early stage (day 11 after induction of differentiation) there were few cells showing lipid droplets in the PLT cultures (Fig. 3A). In contrast in XV medium the majority of the cells contained lipid droplets. Similar results were observed in cultures at both early and expanded passages (Fig. 3A). Analysis of gene expression at early passage (Fig. 3B) showed that the transcription factor *PPAR-gamma* levels were significantly lower in cells cultured in PLT versus cultures in FBS (P < 0.001) and XV medium (P < 0.05). The expression of the mature adipocyte marker *AP2* was significantly higher in XV cultured cells versus FBS (P < 0.05) and PLT cultured cells (P < 0.01). Expression levels in PLT cultured cells were significantly lower versus FBS cultured cells (P < 0.001). Similar results were observed in cells differentiated after expanded passage (Fig. 3C).

**Fig. 4:**
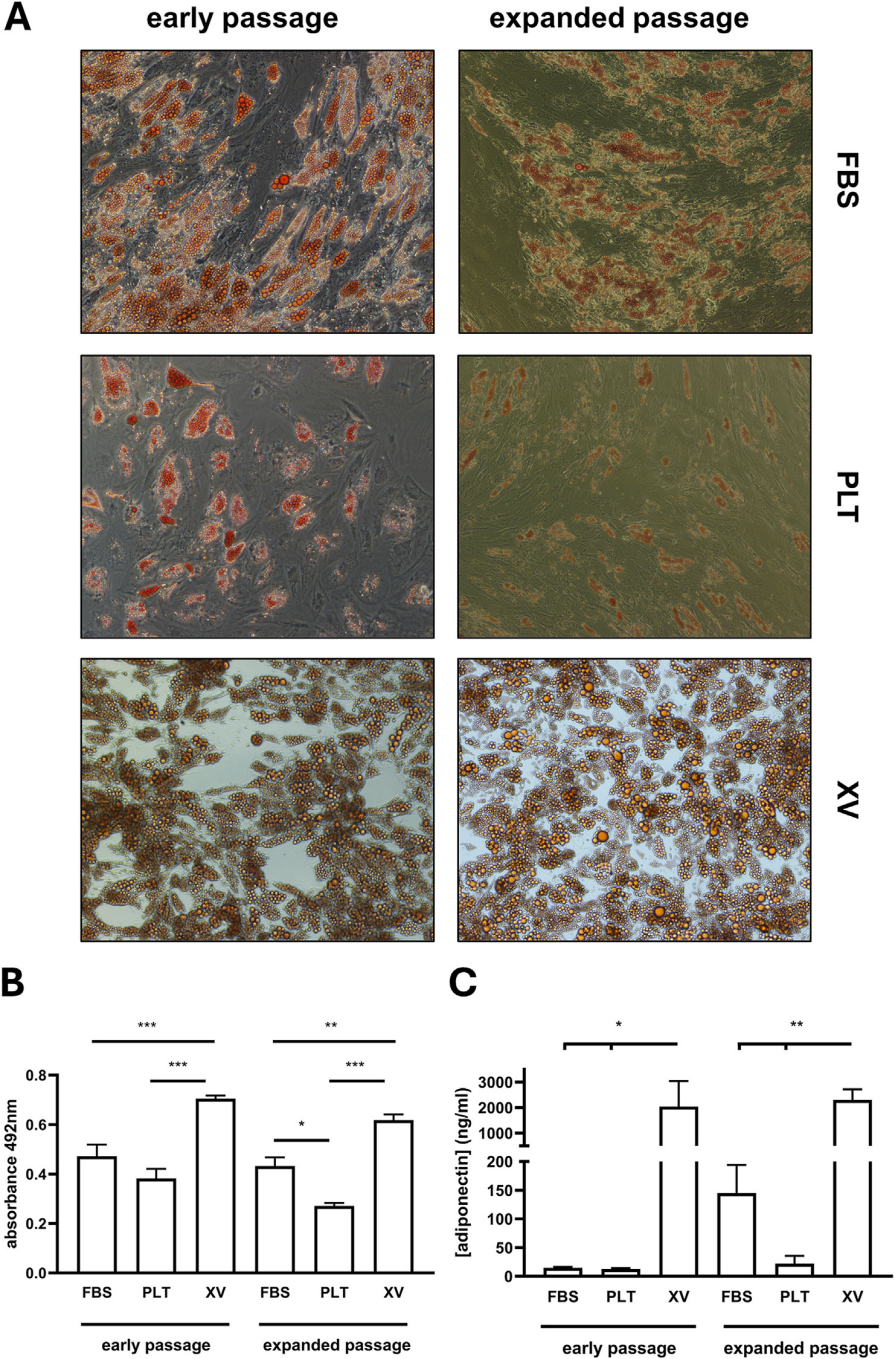
**Adipogenic differentiation of ASCs after 21 days**. **(A)** Images of cells which had been cultured in medium containing FBS or PLT or in Prime-XV MSC Expansion XSFM medium (XV) after exposure to adipogenic differentiation medium and stained with Oil Red O. Representative images are shown from one donor and the experiment was performed on 7 donors in total. **(B)** Quantification of Oil Red O after destaining. **(C)** ELISA analysis of adiponectin secretion by the differentiated cells (n = 4–5 donors) ∗ Represents p < 0.05, ∗∗p < 0.01 and ∗∗∗p < 0.001 significantly different between the groups shown with connecting lines. Scale bar = 50 μm.

**Fig. S2:**
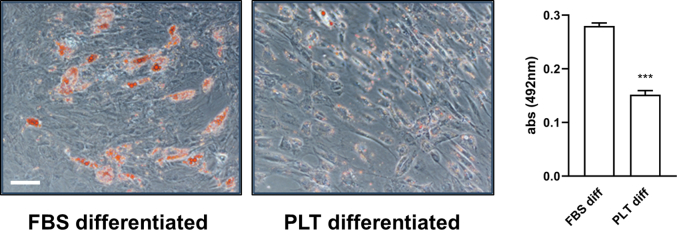
Adipogenic differentiation in the presence of PLT. Cultures of cells expanded in PLT were differentiated under standard conditions in medium containing FBS or in PLT. Oil Red O staining was quantified after 3 weeks (n = 5 donors) ∗∗∗ represents P < 0.001 significant difference between the groups.

Both early and expanded passage cells were stained with Oil Red O after 21 days treatment with adipogenic differentiating factors (Fig. 4). At this time point, compared with day 11, it was clear that now more cells had differentiated in the PLT cultured cells suggesting that these cells might possess delays in the differentiation process. Quantification of Oil Red O staining showed significantly higher values in XV cultured cells versus FBS (P < 0.001) and PLT cultured cells (P < 0.001) in the early passage differentiated cells (Fig. 4A). Similar results were observed in the expanded passage cells (Fig. 4B). In this group the PLT cultured cells showed significantly inferior Oil Red O staining compared with both FBS (P < 0.05) and XV medium (P < 0.001) cultured cells. We investigated whether the impaired results in PLT cultured cells might have been caused by a switch back to differentiating in the presence of FBS. Rather surprisingly we found that PLT cultured cells differentiated in presence of PLT showed even more impaired formation of adipocytes (Supplemental Fig. 2). Consistent with the clearly superior results of adipogenic differentiation of XV cultured cells, ELISA analyses showed significantly higher levels of secreted adiponectin protein in these samples versus FBS (P < 0.05) and PLT (P < 0.05) in the early passage differentiated cells (Fig. 4C). The inferior differentiation in PLT cultured cells was further highlighted in the expanded passage cells showing lower adiponectin secretion versus FBS and XV medium cells (P < 0.01).

### 3.3 Growth factor expression and angiogenesis

**Fig. 5:**
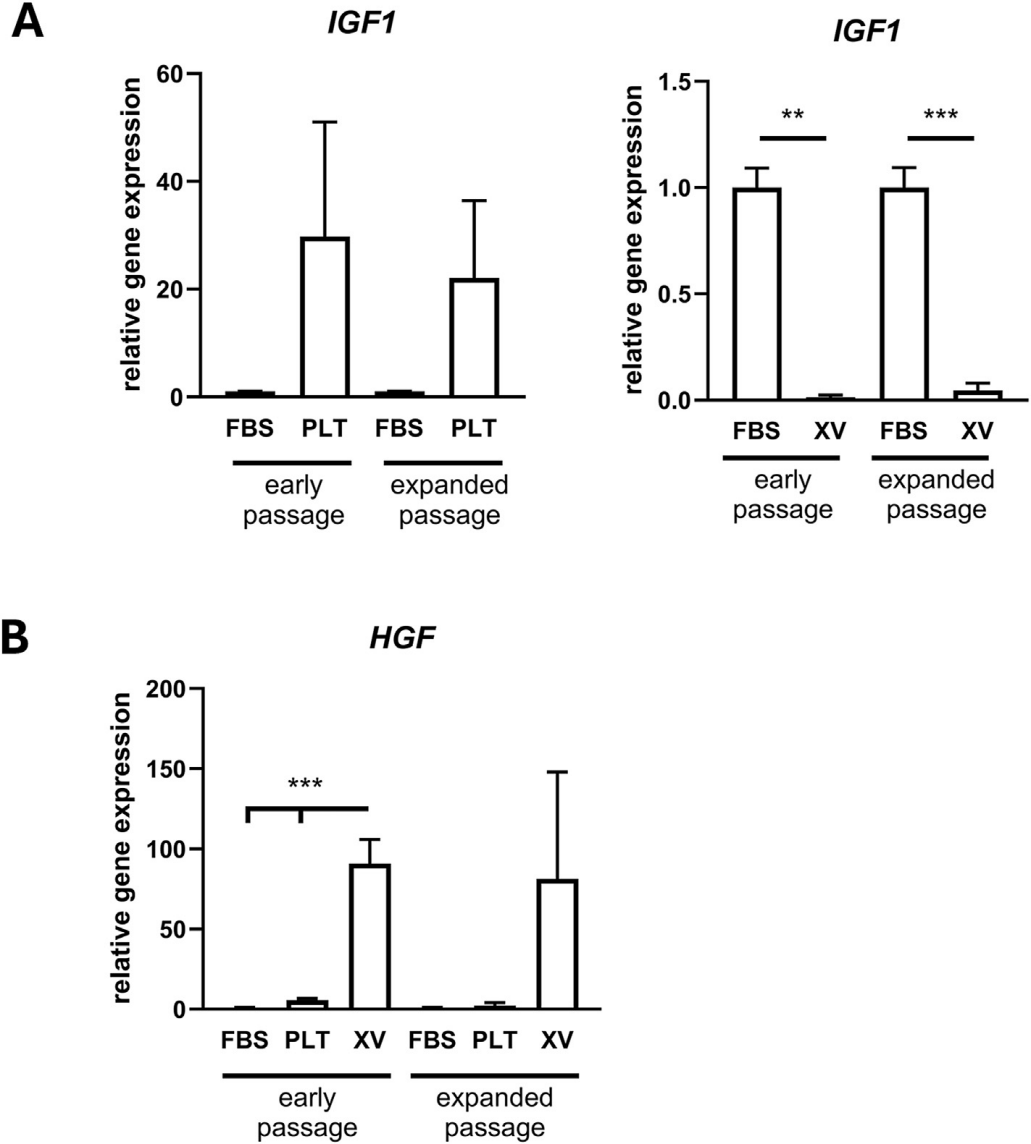
**Expression of growth factors in the ASCs**. qRT-PCR analysis of **(A)***IGF-1* and **(B)***HGF* expression in ASCs cultured in medium containing FBS or PLT or in Prime-XV MSC Expansion XSFM medium (XV). Histograms show mean ± s.e.m from 5 to 7 individual donors. Data are expressed relative to levels in cells cultured in medium containing FBS. ∗P < 0.05; ∗∗P < 0.01; ∗∗∗P < 0.001 significant differences between corresponding different cell culture medium groups.

Expression levels of a variety of angiogenic and pro- and anti-inflammatory genes were analyzed in early and expanded passage ASCs cultured under the three different conditions. The majority of those tested did not show significant differences (Table 2). However, there were notable differences in the expression levels of *IGF1* – compared with FBS cultured cells the PLT cultured cells showed 20-30-fold higher expression and the XV cultured cells showed 20-fold significantly (P < 0.001) lower expression (Fig. 5A). *HGF* expression was 90-fold greater in XV cultured cells compared with FBS cultured cells and there was a more modest 2–6 fold higher expression in PLT cultured cells (Fig. 5B).

**Table 2:** Angiogenic, pro-inflammatory and anti-inflammatory gene expression in ASCs.

Gene	PLT	XV
*ANG*	2.91 ± 0.95	1.25 ± 0.83
*ANGPT1*	1.33 ± 0.12	0.55 ± 0.13
*VEGFA*	1.53 ± 0.25	1.96 ± 0.32
*IL1B*	0.53 ± 0.66	0.23 ± 0.08
*TNF*	0.63 ± 0.21	2.53 ± 0.92
*IL10*	0.98 ± 0.58	1.13 ± 0.70
*MCP1*	0.89 ± 0.26	0.31 ± 0.10
*TGFB1*	2.13 ± 0.73	1.27 ± 0.30

Expression levels (mean ± s.e.m) shown are for cells cultured in PLT or XV medium at early passage versus cells in FBS. There were no significant differences between the groups.

**Fig. 6:**
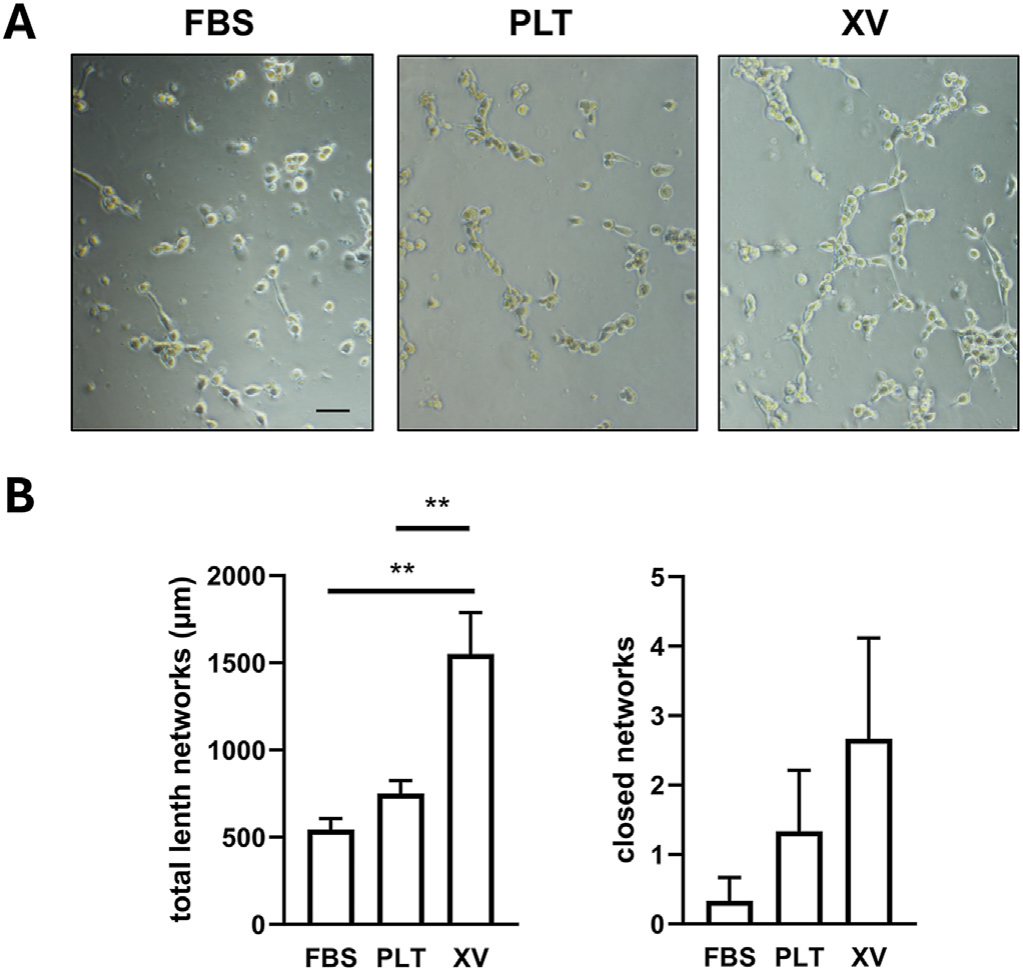
***In vitro*angiogenesis assay. (A)** Endothelial cells seeded on a nutrient-rich matrix were cultured in cell-conditioned medium from FBS, PLT and XV cultured cells. The extent of *in vitro* angiogenesis under the different conditions was assessed by **(B)** quantification of the total length of the tube-like networks and **(C)** the number of closed networks. Histograms show mean ± s.e.m from pooled supernatants from 7 donors. The experiment was repeated twice. ∗∗P < 0.01 significant difference between the groups shown with the connecting lines.

Cell-conditioned medium was tested for biological activity in an *in vitro* angiogenesis assay (Fig. 6A). Endothelial cells (HUVECs) seeded on a nutrient-rich matrix adopt a capillary-like tube network. Compared with FBS and PLT cell-conditioned medium the XV cells-conditioned medium evoked significantly (P < 0.01) greater network extension (Fig. 6B). Furthermore, the number of closed networks was the greatest in the presence of medium from XV cultured cells (Fig. 6B). PLT cultured cell-conditioned medium increased the number of closed networks versus FBS cultured cells (Fig. 6B). Taken together these results suggest that both PLT and XV cultured cells secrete more angiogenic growth factors versus the FBS cultured cells.

## 4 Discussion

Cell-based therapies with ASCs are a valuable tool in various clinical applications due to their excellent regenerative properties. Today more than a hundred clinical trials with ASCs are registered in the US National Institute of Health database (https://www.clinicaltrials.gov/) but still optimal xeno-free culture options are required to ensure safety and increased efficacy. It is important that the procedure for cell isolation and expansion is standardized, and that the cells prepared for transplantation are well characterized. Protocols for harvesting of ASCs and expansion are well described but the culture media varies greatly and still the majority of studies utilise FBS supplementation. It has been identified that use of xeno-free products to avoid the risk of contamination from bacteria, viruses and prions, and to minimise possible allergic reactions is preferred [21]. In this study we investigated ASCs cultured in either GMP-compatible PRIME-XV MSC expansion XSFM (XV) medium or human platelet lysate (PLT) and compared them with cultures in FBS. With a view to determining the suitability of ASCs for transplantation into fat grafts we focussed on the phenotypic properties of the cells, their proliferation and adipogenic differentiation, and growth factor expressions and angiogenic activity when grown under the different culture media conditions. We found that cultures in XV medium proliferated the fastest, expressed the highest number of CD146 positive cells, and showed the best adipogenic and angiogenic properties.

To be able to get a sufficient quantity of cells for transplantation into patients requiring extensive reconstructive surgery there is a need to culture the cells for an extended period of time. After 3 months of expansion the growth curves in all groups plateaued indicating that even if the XV cells expanded very fast initially, they did not acquire tumour cell-like growth properties. The high proliferation capacity and the healthy morphology in late expanded XV-cells are confirmed by earlier studies [17,22]. Heathman et al. [22] previously studied bone marrow stem cells grown in the same xeno-free medium we chose to investigate. Compared with FBS conditions the xeno-free cells had significantly higher proliferation and the results suggested an increased consistency in stem cell characteristics across the donors but there were no flow cytometric analyses done on the cells [22]. In our study we also assessed the proliferation of the ASCs in medium containing 2 % (v/v) PLT which was found to be inferior to XV but similar to levels in 10 % (v/v) FBS. A majority of other studies investigating PLT used concentrations of 5–10 % (v/v) PLT [17,[23], [24], [25], [26], [27]] and this may have influenced the inferior proliferation rates that we observed. In another study, a PLT concentration of 5 % increased growth of the ASCs significantly better than that of 1 %, and slightly but not significantly better than that of 10 % [28].

All three growth conditions produced cultures in which >98 % cells were positive for the typical stem cell markers CD73, CD90 and CD105. In contrast a smaller fraction of the cells were positive for the pericyte marker CD146, which was differentially expressed in the various groups. XV cultures had significantly greater expression of CD146 compared with PLT and FBS, both at early and late passage. This may explain the superior growth rate in the XV cells, and it is consistent with our earlier studies [13,29]. In other reports comparing cells grown in PLT versus FBS the stem cells marker profiles were similar [30,31] as we also observed. A recent study compared two different kinds of GMP classified chemically made xeno-free media, StemPro® and StemMacs®, with FBS [32]. The proliferation rates were increased in the xeno-free medium after prolonged expansion and CD146 expression remained high in StemPro® whereas it decreased with prolonged passaging in FBS. It was also reported that the morphology of the cells in the xeno-free medium looked healthier. In contrast, another study using Wharton Jelly-derived stem cells showed that using FBS as supplement enabled prolonged expression of CD146 compared with the xeno-free medium [33]. However, the xeno-free medium showed enhanced adipogenic differentiation as we also observed. However, Kachroo et al. [34] recently compared chondrogenic stem cells grown in PLT versus FBS and showed that the PLT group had higher proliferation, osteogenic differentiation and expression of CD146 (in contrast with our study).

A number of studies have reported no difference in adipogenic potential between PLT and FBS [30,31], which is in contrast to our study. Taken together with our results it is thus likely that for efficient differentiation 5–10 % PLT is required. Indeed, adipogenesis and osteogenesis, seem to be increased at higher concentrations of PLT (20 % > 10 % > 5 %) [35]. Shansky et al. [28] compared ASCs cultured in FBS and PLT and observed no differences in surface markers whereas PLT-cells had higher adipogenic potential. Another study showed that canine MSCs expanded in different concentrations of PLT and FBS had no differences in proliferation, and no differences in stem cells marker expression but in 10 % PLT the adipogenic and osteogenic potential was increased [36]. Other studies have seen that PLT may be inferior when it comes to chondrogenesis [19]. Diez et al. [37] compared the properties of bone marrow stem cells cultured in supplement derived from human plasma versus FBS. The results showed that there were no differences in proliferation, adipogenic or osteogenic functions between the groups.

In spite of the generally positive results described when using PLT as supplement one problem with using PLT is that the manufacturing of this product is not yet uniformly standardized [38,39]. Another concern with the use of PLT in clinical trials is that it must be treated with either radiation or other pathogen-attenuating processes to eliminate the risk of transmission of potential microbial contaminants. Furthermore, the PLT is commonly used as a mixed pool of human platelets and there is batch-to-batch variance that may lead to different effects in the stem cells cultures [24,40]. Thus, we would argue that a well-defined xeno-free culture medium is preferable to PLT supplement. In this study the XV cells had clearly higher adipogenic potential compared with FBS and PLT, confirmed by gene expression and ELISA. A number of other studies have also concluded that different types of defined xeno-free medium can support efficient adipogenic differentiation [17,[41], [42], [43]].

Our results showed that angiogenic gene expression was variably influenced by the different culture conditions. *HGF* was 90-fold higher in XV cells. HGF can affect angiogenesis through various actions including enhancing proliferation and migration of endothelial cells [44], working synergistically with other growth factors such as VEGF which mediates vessel growth [45] and remodelling of the extracellular matrix [46]. Consistent with this, the XV expanded cells had clearly higher formation of vessel formation in our angiogenic assay, whereas FBS expanded cells were the most inferior group. The high expression of *HGF* in the XV cultures might be explained by the higher percentage of CD146 cells. Furthermore, there was 30-fold higher expression of *IGF1* in the PLT cells and this might contribute to the enhanced angiogenic activity of the PLT cells versus FBS. IGF1 has many similar pro-angiogenic effects as HGF [47]. *IGF1* expression was however 20-fold lower in XV cells versus FBS expanded cells suggesting that the different signalling pathways influencing angiogenesis may be driven by the balance between the levels of these growth factors.

Previous other studies have shown that PLT cultured cells express more growth factors compared with cells under conventional culture conditions. A range of cytokines important for wound healing were shown to be highly secreted from ASCs in PLT groups compared with the FBS groups [48]. Similarly immunomodulatory cytokines and angiogenic factors secreted by infrapatellar fat pad-derived stem cells were also increased under PLT conditions [49]. Although we did not observe significant increases in biological activity in the angiogenic assay with PLT cell-conditioned medium another study has shown that conditioned medium from PLT-cultured ASCs-sheets significantly enhanced tube formation of endothelial cells [50]. Interestingly this effect was in part mediated by HGF. However, another study showed no differences between FBS and PLT-cultured ASCs with respect to the ability to form closed vessel like structures even though the PLT cell-conditioned medium contained higher vascular endothelial growth factor levels [51]. Consistent with our results, another study described that Wharton's jelly-derived stem cells cultured in another manufacturer's xeno-free medium promoted longer endothelial tube structures compared with FBS conditions [52]. Furthermore, that effect was reduced in the presence of HGF blocking antibody. Sagaradze et al. [53] also showed that culture in another xeno-free medium formulation was associated with enhanced levels of HGF and angiopoietin-1 although vascular endothelial growth factor was higher in FBS cultures. Collectively, our study and the others suggest that stem cell culture in FBS medium might impair the expression of HGF.

To summarise, in this study we have shown that XV medium expanded cultures had higher percentage of CD146 positive cells and this might account for significantly higher proliferation and higher differentiation capacity towards adipocytes and enhanced angiogenic activity, which are important aspects for helping fat graft survival in patients undergoing reconstructive surgery. These biological functional effects were our primary focus of investigation but our screen of a range of angiogenic, pro- and anti-inflammatory genes also indicated that *HGF* and *IGF1* expression levels were most influenced by the culture conditions. Further studies utilising RNA sequencing could be performed to capture a more comprehensive profile of gene expression and subsequent gene ontology and pathway enrichment analyses undertaken to interpret the potential functional significance of any differentially expressed genes.

## Credit author statement

Anne Therese Lauvrud: methodology, formal analysis, writing the original draft manuscript, and review & editing the final version of the manuscript. Maria Vittoria Giraudo: methodology and formal analysis. Rebecca Wiberg: funding for the study and supervision. Mikael Wiberg: funding for the study. Paul J Kingham: conception of the study, methodology, formal analysis, funding for the study, supervision and review & editing final version of the manuscript. Maria Brohlin: conception of the study, methodology, funding for the study, supervision and review & editing final version of the manuscript. All authors have read and agreed to the ﬁnal submitted version of the paper.

## Ethics approval

This work described has been carried out in accordance with The Code of Ethics of the World Medical Association (Declaration of Helsinki) for experiments involving humans. Informed consent was obtained from the donors and procedures were approved by the Local Ethical Committee for Clinical Research in Umeå University (2013-276-31M).

## Data availability

Data will be made available on request.

## Funding

This work was financially supported by Forskningslyftet, Medical Faculty Umeå University, Västerbotten County Council and jointly through a regional agreement with Umeå University medical faculty (ALF) and Vinnova project CAMP (Contract no. 2017–02130).

## Declaration of Generative AI and AI-assisted technologies in the writing process

No disclosures.

## Declaration of competing interest

Authors declare no competing interest.

## Footnotes

Appendix A Supplementary data to this article can be found online at https://doi.org/10.1016/j.reth.2024.09.013.
